# A One Health investigation of *Salmonella enterica* serovar Wangata in north-eastern New South Wales, Australia, 2016–2017

**DOI:** 10.1017/S0950268819000475

**Published:** 2019-03-12

**Authors:** J. Collins, K. M. J. Simpson, G. Bell, D. N. Durrheim, G. A. Hill-Cawthorne, K. Hope, P. Howard, T. Kohlenberg, K. Lawrence, K. Lilly, P. Porigneaux, V. Sintchenko, Q. Wang, M. P. Ward, A. Wiethoelter, S. M. Mor, J. Flint

**Affiliations:** 1 Hunter New England Population Health, Wallsend, NSW, Australia; 2 National Centre for Epidemiology and Population Health, Australian National University, Canberra, ACT, Australia; 3 School of Veterinary Science, Faculty of Science, The University of Sydney, Sydney and Camden, NSW, Australia; 4 North Coast Public Health, Lismore and Port Macquarie, NSW, Australia; 5 School of Medicine and Public Health, University of Newcastle, NSW, Australia; 6 College of Public Health, Medical and Veterinary Sciences, James Cook University, QLD, Australia; 7 Marie Bashir Institute for Infectious Diseases and Biosecurity, The University of Sydney, Sydney, NSW, Australia; 8 School of Public Health, The University of Sydney, Sydney, NSW, Australia; 9 Health Protection New South Wales, Sydney, NSW, Australia; 10 NSW Enteric Reference Laboratory, Centre for Infectious Diseases and Microbiology Laboratory Services, Pathology West, Sydney, NSW, Australia; 11 Centre for Infectious Diseases and Microbiology-Public Health, Westmead Hospital, Sydney, NSW, Australia; 12 Sydney Medical School, The University of Sydney, Sydney, NSW, Australia; 13 Faculty of Veterinary and Agricultural Sciences, University of Melbourne, Melbourne, VIC, Australia

**Keywords:** One Health, outbreaks, salmonellosis, whole genome sequencing, zoonoses

## Abstract

*Salmonella enterica* serovar Wangata (*S*. Wangata) is an important cause of endemic salmonellosis in Australia, with human infections occurring from undefined sources. This investigation sought to examine possible environmental and zoonotic sources for human infections with *S*. Wangata in north-eastern New South Wales (NSW), Australia. The investigation adopted a One Health approach and was comprised of three complimentary components: a case–control study examining human risk factors; environmental and animal sampling; and genomic analysis of human, animal and environmental isolates. Forty-eight human *S.* Wangata cases were interviewed during a 6-month period from November 2016 to April 2017, together with 55 *Salmonella* Typhimurium (*S.* Typhimurium) controls and 130 neighbourhood controls. Indirect contact with bats/flying foxes (*S*. Typhimurium controls (adjusted odds ratio (aOR) 2.63, 95% confidence interval (CI) 1.06–6.48)) (neighbourhood controls (aOR 8.33, 95% CI 2.58–26.83)), wild frogs (aOR 3.65, 95% CI 1.32–10.07) and wild birds (aOR 6.93, 95% CI 2.29–21.00) were statistically associated with illness in multivariable analyses. *S.* Wangata was detected in dog faeces, wildlife scats and a compost specimen collected from the outdoor environments of cases’ residences. In addition, *S.* Wangata was detected in the faeces of wild birds and sea turtles in the investigation area. Genomic analysis revealed that *S.* Wangata isolates were relatively clonal. Our findings suggest that *S*. Wangata is present in the environment and may have a reservoir in wildlife populations in north-eastern NSW. Further investigation is required to better understand the occurrence of *Salmonella* in wildlife groups and to identify possible transmission pathways for human infections.

## Introduction

*Salmonella enterica* is an important cause of gastrointestinal illness in humans and can be transmitted through food, water, animals, the environment and person-to-person [1]. There are approximately 2500 different serovars (or serotypes) of *Salmonella*, each with distinct geographical and epidemiological characteristics [2]. The majority of human infections with *Salmonella* in Australia have been attributed to foodborne transmission pathways [3]. This is largely driven by the predominance of *Salmonella* serovar Typhimurium (*S*. Typhimurium), which has commonly been associated with eggs in outbreak investigations [1]. Environmental and zoonotic transmission pathways also play a distinct role in human *Salmonella* infections, particularly in sub-regions of the country where climatic and environmental variations appear to have permitted ecological niches for specific serovars to be established [1, 4, 5]. *Salmonella* outbreaks in Tasmania, New South Wales (NSW) and the Northern Territory have previously been attributed to indirect exposure to native animals and animal faeces [6–8].

Notifications of human infections with *Salmonella* serovar Wangata (*S*. Wangata) have progressively increased in NSW over the past decade, from a rate of 0.43 per 100 000 in 2006 to 1.34 per 100 000 in 2016 (unpublished data, NSW Notifiable Conditions Information Management System, 2006–2016). The majority of infections are notified from the north-eastern region of NSW, where *S*. Wangata is the second highest notified serovar after *S.* Typhimurium. Notifications of *S*. Wangata remain low in other parts of the state, indicating a possible ecological niche in this region. Notified cases of *S.* Wangata are routinely investigated by public health staff in NSW. No common exposures were identified in case interviews from 2011 to 2015 (unpublished data, OzFoodNet, Hunter New England Population Health). The geographical distribution of cases and the lack of common food exposures suggested that transmission of this serovar was more likely to be environmental or zoonotic, rather than foodborne.

We conducted an outbreak investigation in north-eastern NSW from November 2016 to April 2017 to elucidate possible environmental and zoonotic transmission pathways for *S.* Wangata infections. We established a One Health investigation team consisting of human health, animal health and laboratory experts. We incorporated three elements into the design of our investigation: a case–control study to identify human risk factors for infection; animal and environmental sampling to determine if *S*. Wangata was present in the environment; and genomic analysis to explore the relatedness of human, animal and environmental isolates.

## Methods

### Case–control study

#### Selection of cases

Cases of *S*. Wangata were identified from the NSW Notifiable Conditions Information Management System (NCIMS). An eligible case was defined as a person residing in the NSW Local Health Districts (LHDs) of Hunter New England, Mid North Coast and Northern NSW with a laboratory-confirmed *S*. Wangata infection and a stool specimen collection date between 1 November 2016 and 30 April 2017. These LHDs were selected as they captured the majority (63%) of *S.* Wangata cases notified in NSW between 2011 and 2015. Cases were excluded from the investigation if they: were unable to be contacted after six attempts, resided in an institution, required an interpreter, travelled more than 100 km from their home during their incubation period (7 days prior to illness onset), could not recall the date their illness began, had a household member with diarrhoea during their incubation period (possible secondary case) or if another enteric pathogen was detected in their stool specimen (co-infection).

#### Selection of controls

Two control groups were used for this investigation. The first was based on a case–case methodology and consisted of persons with a notified *S.* Typhimurium infection. *S*. Typhimurium controls were identified from NCIMS and were frequency-matched to cases by age group (0–4, 5–14, 15–64 and ⩾65 years). The second control group consisted of neighbourhood controls that were frequency-matched to cases by geographic proximity. Using publicly available geocoded national address files [9] and ArcGIS mapping software (ArcGIS Desktop Version 10.5, Environmental Systems Research Institute, Redlands: CA, USA), a 2 km radius was drawn around a case's residence and all addresses were extracted and exported to Microsoft Excel 2016 (Microsoft Corporation, Redmond: Washington, USA). Where the number of extracted addresses was <200, the process was repeated using a 5 km radius. A random number generator [10] was used to randomly select 20 addresses within the neighbourhood of each case. A participant letter and questionnaire were mailed to the households selected. Households were asked to complete the questionnaire for the person in the household who had the next birthday.

#### Data collection

Cases and *S*. Typhimurium controls were interviewed via telephone by public health staff. Neighbourhood controls completed a self-administered questionnaire either by mail (reply paid) or online using Select Survey (ClassApps, Kansas City: MO, USA).

Data were collected on environmental and zoonotic exposures in the 7 days prior to illness onset (or in the last 7 days for neighbourhood controls) and included: property size, private drinking water, home-grown foods, contact with soil or grass at home (such as gardening), outdoor activities (such as visiting a park/playground, national park or swimming in natural waterways) and contact with animals. Direct and indirect exposure to household pets, livestock and wildlife were captured and measured using the following criteria: direct contact was defined as touching or patting the animal or having direct contact with animal faeces, whereas indirect contact was defined as being in the same environment as the animal without direct contact.

Demographic information, such as age, sex and location, was collected from NCIMS for cases and *S*. Typhimurium controls. Neighbourhood controls were asked to identify their age group. Cases and controls were classified as either urban (major cities, inner regional) or rural (outer regional, remote, very remote) based on the Australian Standard Geographical Classification (ASGC) Remoteness Areas (2006) [11].

#### Data analysis

Crude odds ratios (ORs) and 95% confidence intervals (CIs) were calculated using univariable logistic regression. Variables were selected for inclusion in a fixed-effects, multivariable model based on statistical significance at *P* < 0.25 in univariable analyses. An initial ‘full’ multivariable model was constructed. Non-statistically significant variables (*P* > 0.05) were removed sequentially from the model using a backward stepwise approach. Likelihood ratio tests were used to determine whether the removed variable was significantly contributing to the model (*P* < 0.05). The final, main-effects model included significantly contributing variables, along with potential confounders (age, rural locality and sex where available). Plausible interaction terms were examined. Model fit was assessed using the Hosmer–Lemeshow and Pearson goodness-of-fit tests, the area under the receiver operating characteristic curve and by examining model residuals.

Fixed-effects, multivariable logistic regression models were constructed for each control group separately. Neighbourhood control data were also analysed using a random-effects model in order to detect any geographic clustering in the data. Questionnaire data were entered and managed in REDCap [12]. Data were analysed using Stata version 14.1 (StataCorp LP, College Station, TX, USA).

### Environmental and animal sampling

#### Cases’ residences

Environmental samples were collected from the outdoor areas of cases’ residences who provided consent during interviews and who were identified as having either an outdoor environment (as opposed to an apartment building) or pets present. Target samples included water, soil and voided animal faecal samples from pets, livestock and wildlife. Samples were collected using faecal specimen jars and were individually bagged to prevent cross-contamination. Samples were sent to the University of Sydney for culture within 24 h and were kept chilled during transport.

#### Wildlife rehabilitation centres

Wildlife in rehabilitation centres within the same geographical region as human cases were sampled. The use of wildlife-in-care as a proxy for pathogen surveillance of free ranging populations has been previously illustrated [13]. Swabs were used to collect faecal specimens either by direct swabbing of the animal or by collecting freshly voided faeces. Samples were sent to the University of Sydney for culture.

### Laboratory analysis

#### Salmonella isolation and serotyping

All human isolates with a presumptive *Salmonella* isolation by pathology service providers in NSW are confirmed by the NSW Enteric Reference Laboratory at the Centre for Infectious Diseases and Microbiology Laboratory Services (CIDMLS), NSW Health Pathology at Westmead [14]. Isolates underwent confirmatory testing and then were serotyped using the White–Kauffmann–Le Minor scheme [2].

For each environmental sample, a sterile swab was used to probe the sample until the swab tip was coated. Swab tips were then broken off into buffered peptone water (Interpath Services, Heidelberg West: VIC, Australia) for pre-enrichment and incubated at 37 °C for 18 ± 2 h. A 0.1 ml of solution was transferred to 9.9 ml of Rappaport Vassilidas Broth (Merck, Macquarie Park: NSW, Australia) and incubated at 42 °C 18 ± 2 h. The broth was streaked onto Xylose Lysine Desoxycholate (XLD) agar (Edwards, Narellan: NSW, Australia) and incubated for 18 ± 2 h at 37 °C. Individual suspect colonies were streaked onto Brilliant Green agar (BGA) (Edwards, Narellan: NSW, Australia) and incubated for 18 ± 2 h at 37 °C. Positive colonies were then confirmed using a RapID biochemical ID test (Thermo Fisher Scientific, Scoresby: VIC, Australia). If positive, an individual colony was subcultured onto nutrient agar (Edwards, Narellan: NSW, Australia) and forwarded to CIDMLS for serotyping using the White–Kauffmann–Le Minor scheme [2].

#### DNA sample preparation

For all *S.* Wangata isolates, a single colony was subcultured on a blood agar plate at 37 °C for 24 h. The fresh colonies were collected and suspended in PBS and proceeded to genomic DNA extraction using a manual extraction kit (Presto™ Mini gDNA Bacteria Kit, Geneaid, Taiwan) following the manufacturer's instructions. DNA quality was checked through an Allsheng Nano-300™ Micro Spectrophotometer (HangZhou Allsheng Instrument CO., LTD, Hangzhou: Zhejiang, China). The DNA concentration was measured using a Quant-iT™ PicoGreen™ dsDNA Assay Kit (Thermo Fisher Scientific, Scoresby: VIC, Australia) on a fluorescence reader (Victor plate reader, PerkinElmer, Waltham: MA, USA).

#### Genome sequencing

A 150 bp paired-end library was prepared for each extracted genomic DNA sample using the Nextera XT library prep kit and the Index set (Illumina, Scoresby: VIC, Australia). Quality control of the libraries was performed by assessing library size distribution on an Agilent 2200 Tapestation (Agilent Technologies, Santa Clara: CA, USA) with fragment sizes ranged between 250 and 1000 bp and libraries were quantified by real-time PCR using a KAPA library quantification kit according to the manufacturer's protocol (Roche, Indianapolis: IN, USA). The 150 bp paired-end reads were generated using a Nextseq500 platform (Illumina) at CIDMLS and the Centre for Infectious Diseases and Microbiology – Public Health (CIDM-PH), NSW Health Pathology at Westmead. The sequence data quality was checked using FastQC (https://www.bioinformatics.babraham.ac.uk/projects/fastqc).

#### Single nucleotide polymorphism analysis

Sample reads were trimmed using Trimmomatic 0.36 [15] to remove trail end bases with a phred score <33. The reads from each isolate were aligned against the core genes of *Salmonella* [16] using Snippy 3.1 (https://github.com/tseemann/snippy). A maximum-likelihood single nucleotide polymorphism (SNP) tree was built using FastTree 2.1.9 [17], which estimates pairwise distances using the Jukes–Cantor model. The Shimodaira–Hasegawa test was used on all three alternate topologies around each split, using the CAT approximation and 1000 resamples.

In order to assess the diversity across the whole genome, a reference *S*. Wangata genome was assembled. The sample reads were imported into CLC Genomics Workbench 10.1.1 (CLC bio, Aarhus, Denmark) and assessed for quality. The isolate with the highest performing quality parameters (16-SWA-004) was selected for *de novo* assembly which was performed using the *de novo* assembly tool in CLC. The resulting contigs were ordered in ABACAS [18] using *S*. Sloterdijk ATCC 15791 as a reference (NCBI GenBank Accession No. CP012349.1) which was selected based on a BLAST search of the larger contigs. The output from ABACAS was compared back to the reference genome using ACT [19] and was visually inspected for ordering errors. The ordered contigs were then joined using 12 iterations of IMAGE [20]. Annotations from *S*. Sloterdijk were transferred to the *S*. Wangata reference using RATT [21]. This sequence was then used as a reference for read mapping and SNP calling and a second SNP tree was built as described above. SNP differences were calculated using snp-dists 0.6 (https://github.com/tseemann/snp-dists).

### Ethics approval

This investigation was approved by the Hunter New England Local Health District Human Research Ethics Committee (LNR/16/HNE/485, granted 1 November 2016), with additional site-specific approval from the Mid North Coast, Northern NSW and Western Sydney LHDs. Human ethics approval was also granted from the Australian National University (2016/605, granted 2 December 2016). Animal ethics approval was granted from the University of Sydney (2016/1076, granted 16 November 2016).

## Results

### Case–control study

In total, 76 cases of *S*. Wangata infection were notified during the investigation period. Of these cases, 48 were eligible to participate and were enrolled. Twelve cases were unable to be contacted and 16 cases met one or more exclusion criteria. No case refused to participate. Seventy *S*. Typhimurium controls were identified during the same period. Fifty-seven *S*. Typhimurium controls were eligible to participate (81%) and 55 agreed to participate (96%). We had a response rate of 20% (213/1040) for neighbourhood controls. Of those who responded, 61% (130/213) were eligible to participate and were enrolled.

The median age of *S*. Wangata cases was 55 years (range 3 months–88 years). Cases were evenly distributed by sex (Table 1). Age groups varied between control groups, with *S*. Typhimurium controls having a younger median age while neighbourhood controls tended to be older (Table 1). Cases were no more likely to live in rural areas compared with controls (Table 1). The majority of *S*. Wangata infections were notified from the Northern NSW LHD (65%). A slightly higher proportion of *S*. Wangata cases were hospitalised (42%) compared with *S*. Typhimurium controls (25%); however, this was not statistically significant (*χ*^2^ = 3.05, df = 1, *P* 0.081) (Table 1).

**Table 1.** Demographic characteristics of *S.* Wangata cases and control groups, north-eastern New South Wales, November 2016–April 2017

	*S*. Wangata cases		*S*. Typhimurium controls		Neighbourhood controls^a^	
	No.	%	No.	%	No.	%
Age (years)						
Median (range)	55 (3m^b^–88)		29 (2–83)		–	–
0–4	11	23	11	20	2	2
5–14	3	6	6	11	4	3
15–64	17	35	31	56	64	49
⩾65	17	35	7	13	60	46
Sex						
Male	26	54	31	56	–	–
Female	22	46	24	44	–	–
Local Health District						
Hunter New England	6	13	33	60	9	7
Mid North Coast	11	23	8	15	26	20
Northern NSW	31	65	14	25	95	73
Location						
Urban	36	75	40	73	98	75
Rural	12	25	15	27	32	25
Hospitalisation						
Hospitalised	20	42	14	25	–	–
Median length of stay (days)	4.5 (1–10)		2 (1–6)		–	–
TOTAL	48		55		130	

a Neighbourhood controls asked to report age group only.

b Three months

#### Univariable analysis

The results from univariable analyses are shown in Table 2. While data on direct and indirect contact with animals were collected, direct contact results are not presented due to low numbers (⩽15%) of cases and controls reporting direct contact with animals (with the exception of pet cats and dogs).

**Table 2.** Univariable logistic regression of exposures associated with *S.* Wangata infection, north-eastern New South Wales, November 2016–April 2017

	Cases (*n* = 48)		*S*. Typhimurium controls (*n* = 55)					Neighbourhood controls (*n* = 130)				
Variables	No.	%	No.	%	OR	95% CI	*P* value	No.	%	OR	95% CI	*P* value
*Property exposures*												
Property size												
1/4 acre or less	31	65	36	65	*Reference*			83	64	*Reference*		
>1/4 acre to 5 acres	10	21	7	13	1.66	0.56–4.88	0.358	21	16	1.27	0.54–3.01	0.579
>5 acres	7	15	12	22	0.68	0.24–1.93	0.467	24	18	0.78	0.31–1.99	0.605
Private water supply	17	35	22	40	0.82	0.37–1.83	0.633	45	35	1.01	0.51–2.02	0.974
Grow fruit trees	22	46	19	35	1.60	0.72–3.55	0.244	63	48	0.87	0.45–1.70	0.689
Grow nut trees	4	8	6	11	0.74	0.20–2.80	0.660	22	17	0.43	0.14–1.33	0.145
Grow vegetables or herbs	23	48	23	42	1.28	0.59–2.79	0.535	73	56	0.69	0.36–1.35	0.281
Ate home-grown food	9	19	9	16	1.22	0.44–3.40	0.701	57	44	0.31	0.14–0.69	0.004
*Outdoor activities*												
Contact with soil or grass	28	58	34	62	1.01	0.90–1.13	0.876	96	74	1.01	0.98–1.04	0.624
Visited park or playground	16	33	21	38	0.79	0.35–1.78	0.564	21	16	2.56	1.19–5.49	0.016
Visited national park/reserve	4	8	4	7	1.09	0.26–4.63	0.906	14	11	0.73	0.23–2.33	0.592
Contact with natural waterways	13	27	19	35	0.68	0.29–1.60	0.380	32	25	1.09	0.51–2.32	0.820
Swam in a pool	10	21	15	27	0.72	0.29–1.80	0.484	14	11	2.14	0.88–5.23	0.094
*Pet contact*												
Pet dog^a^												
Direct	30	63	37	67	0.76	0.32–1.78	0.529	62	48	1.54	0.76–3.14	0.232
Indirect	2	4	3	5	0.63	0.09–4.28	0.632	10	8	0.64	0.13–3.22	0.586
Pet cat^a^												
Direct	12	25	16	29	0.89	0.36–2.20	0.799	27	21	1.27	0.56–2.85	0.566
Indirect	9	19	5	9	2.13	0.64–7.13	0.219	5	4	5.13	1.58–16.67	0.006
Pet bird	5	10	4	7	1.46	0.37–5.81	0.588	10	8	1.02	0.33–3.19	0.967
Pet chicken	5	10	8	15	0.57	0.17–1.88	0.352	10	8	1.04	0.33–3.22	0.952
Pet fish	2	4	5	9	0.43	0.08–2.31	0.323	9	7	0.42	0.09–2.05	0.286
Pet turtle	0	0	0	0	–	–	–	0	0	–	–	–
Pet snake	0	0	0	0	–	–	–	0	0	–	–	–
Pet lizard	0	0	1	2	–	–	–	1	1	–	–	–
*Livestock contact*												
Cow	5	10	8	15	0.64	0.19–2.12	0.465	8	6	1.60	0.49–5.18	0.432
Sheep	0	0	3	5	–	–	–	1	1	–	–	–
Horse	4	8	1	2	4.65	0.50–43.21	0.176	1	1	10.23	1.11–94.17	0.040
Goat	0	0	1	2	–	–	–	1	1	–	–	–
Alpaca	0	0	0	0	–	–	–	13	10	–	–	–
Pig	0	0	2	4	–	–	–	1	1	–	–	–
Chicken	1	2	2	4	0.53	0.05–6.06	0.611	13	10	0.17	0.02–1.30	0.087
*Wildlife contact*												
Kangaroo	13	27	13	24	1.20	0.49–2.92	0.688	13	10	2.94	1.25–6.95	0.014
Quoll	2	4	0	0	–	–	–	0	0	–	–	–
Possum	8	17	13	24	0.62	0.23–1.65	0.333	9	7	2.36	0.85–6.53	0.100
Bandicoot	9	19	8	15	1.33	1.47–3.79	0.591	8	6	3.20	1.15–8.88	0.026
Ibis	16	33	7	13	3.43	1.27–9.27	0.015	14	11	3.68	1.62–8.35	0.002
Seagull	10	21	7	13	1.80	0.63–5.19	0.273	4	3	7.37	2.18–24.87	0.001
Duck	16	33	16	29	1.22	0.53–2.81	0.643	18	14	2.75	1.26–6.01	0.011
Wild bird	38	79	42	76	1.11	0.41–2.96	0.841	28	22	12.67	5.45–29.43	<0.001
Bat/flying fox	30	63	21	38	2.86	1.26–6.48	0.012	18	14	10.21	4.64–22.45	<0.001
Wild frog	23	48	15	27	2.39	1.05–5.44	0.038	21	16	4.03	1.93–8.43	<0.001
Wild snake	15	31	16	29	1.08	0.46–2.51	0.859	10	8	4.50	1.84–10.98	0.001
Wild lizard	32	67	25	45	2.24	1.00–5.02	0.050	18	14	10.33	4.72–22.64	<0.001

OR, crude odds ratio; CI, confidence interval.

Variables with *P* < 0.25 in univariable analysis were included in multivariable models.

a Direct contact results shown for pet dogs and cats only. For all other animals, indirect contact only is shown as direct contact was low (⩽15%).

Property exposures and outdoor activities were not generally identified as being associated with *S.* Wangata infection in univariable analyses. The only association that was statistically significant (*P* < 0.05) was visiting a park or playground when compared with neighbourhood controls (Table 2). Similarly, pet contact was not generally associated with *S.* Wangata infection, with the exception of indirect contact with pet cats which was statistically significant when compared with neighbourhood controls. Although direct contact with pet dogs was high among cases and both control groups (63%, 67% and 48%, respectively), it was not significantly associated with illness. No cases reported contact with pet reptiles (turtles, lizards or snakes) during their incubation period (Table 2). Contact with livestock was low. The only livestock association with *S*. Wangata infection that was statistically significant was indirect contact with horses when compared with neighbourhood controls (Table 2).

There were a number of significant associations (*P* < 0.05) with indirect contact with wildlife in univariable analyses. Indirect contact with ibises, bats/flying foxes, wild frogs and wild lizards were significantly associated with illness when compared with *S*. Typhimurium controls. Indirect contact with kangaroos, bandicoots, ibises, seagulls, ducks, wild birds, bats/flying foxes, wild frogs, wild snakes and wild lizards were significantly associated with illness (*P* < 0.05) when compared with neighbourhood controls (Table 2).

#### Multivariable analysis

Exposure variables that had a *P* value < 0.25 in univariable analyses were included in separate multivariable models by control group, together with confounders. Exposure variables that significantly contributed to the final multivariable models are shown in Table 3. Indirect exposure to wildlife was significant in both multivariable models. Specifically, we found that the odds of indirect exposure to bats/flying foxes and wild frogs was higher among *S.* Wangata cases compared with *S.* Typhimurium controls. When compared with neighbourhood controls, *S*. Wangata cases had a higher odds of indirect exposure to bats/flying foxes and wild birds. The odds of direct exposure to pet chickens was lower among *S.* Wangata cases compared with *S*. Typhimurium controls. In addition, the odds of exposure to growing nut trees or eating home-grown foods was lower among *S.* Wangata cases compared with neighbourhood controls.

**Table 3.** Multivariable logistic regression models of exposures associated with *S.* Wangata infection, north-eastern New South Wales, November 2016–April 2017

Exposure variable	aOR	95% CI	*P* value
Model 1: *S*. Typhimurium controls			
Pet chicken (direct contact)	0.09	0.01–0.93	0.043
Pet chicken (indirect contact)	0.57	0.15–2.17	0.409
Bat/flying fox (indirect contact)	2.63	1.06–6.48	0.036
Wild frog (indirect contact)	3.65	1.32–10.07	0.012
Model 2: neighbourhood controls			
Grow nut trees	0.08	0.01–1.00	0.050
Ate home grown food	0.30	0.09–1.00	0.050
Bat/flying fox (indirect contact)	8.33	2.58–26.83	<0.001
Wild bird (direct contact)	1.50	0.12–19.20	0.755
Wild bird (indirect contact)	6.93	2.29–21.00	0.001

aOR, adjusted odds ratio; CI, confidence interval

### Environmental and animal sampling

Of the 48 cases, 24 (50%) agreed to environmental sampling and met the sampling criteria. *S*. Wangata was isolated from samples collected from the outdoor environment of four cases’ residences. A summary of the environmental sample results is provided in Table 4. Due to the time required for cases’ illness presentation, specimen testing and notification to health departments, there was a delay between cases’ illness onset (diarrhoea) and the date that environmental samples were collected. The median time between cases’ onset of illness and sample collection was 31 days (range 18–59 days). For the four cases where *S.* Wangata was found in cases’ outdoor environments, the median time between illness onset and sample collection was 34.5 days (range 28–42 days).

**Table 4.** Salmonella isolated from the outdoor environment at cases’ residences

Sample type	No. samples	No. positive	Serovar (No. isolated)
Environment			
Compost	2	2	*S*. Wangata (1), *S*. Kiambu (1)
Soil/leaf litter	58	2	*S*. Birkenhead (2)
Food	1	0	
Fruit/tree nut	14	0	
Sand	2	0	
Water	46	0	
Faeces			
Companion	30	2	*S.* Wangata (1^a^), *S.* Birkenhead (1^b^)
Livestock	5	0	
Wildlife	24	3	*S.* Wangata (3^c^)
TOTAL	182	9	

a Dog (W27).

b Dog (W45).

c Suspected bandicoot (W07), suspected brush turkey (W36), suspected wild bird (W36)

Of the 14 wildlife rehabilitation centres approached by the investigation team, nine participated by submitting samples. Of the 48 samples received, *S*. Wangata was isolated in samples submitted by two of the wildlife rehabilitation centres, yielding a total of four positive animals: two green sea turtles (*Chelonia mydas*), an Australian pelican (*Pelecanus conspicillatus*) and a black swan (*Cygnus atratus*) (Table 5). A detailed list of wildlife species sampled during the investigation is provided in Appendix 1.

**Table 5.** *Salmonella* isolated from wildlife in rehabilitation centres

Sample origin	No. species	No. samples	No. positive	Serovar (No. isolates)
Mammals	8	23	2	*S.* Bovismorbificans (1^a^), *S*. Kottbus (1^b^)
Birds	11	17	4	*S*. Wangata (2^c^,^d^), *S*. Bovismorbificans (1^e^), *S*. Chailey 1^f^)
Reptiles	5	8	2	*S*. Wangata (2^g^)
TOTAL	24	48	8	

a Gould's long-eared microbat (*Nyctophilus gouldi*).

b Common wombat (*Vombatus ursinus*).

c Australian pelican (*Pelecanus conspicillatus*).

d Black swan (*Cygnus atratus*).

e Magpie (*Gymnorhina tibicen*).

f Tawney frogmouth (*Podargus strigoides*).

g Green sea turtle (*Chelonia mydas*).

### Phylogenomic analysis of human, animal and environmental isolates

**Fig. 1.**
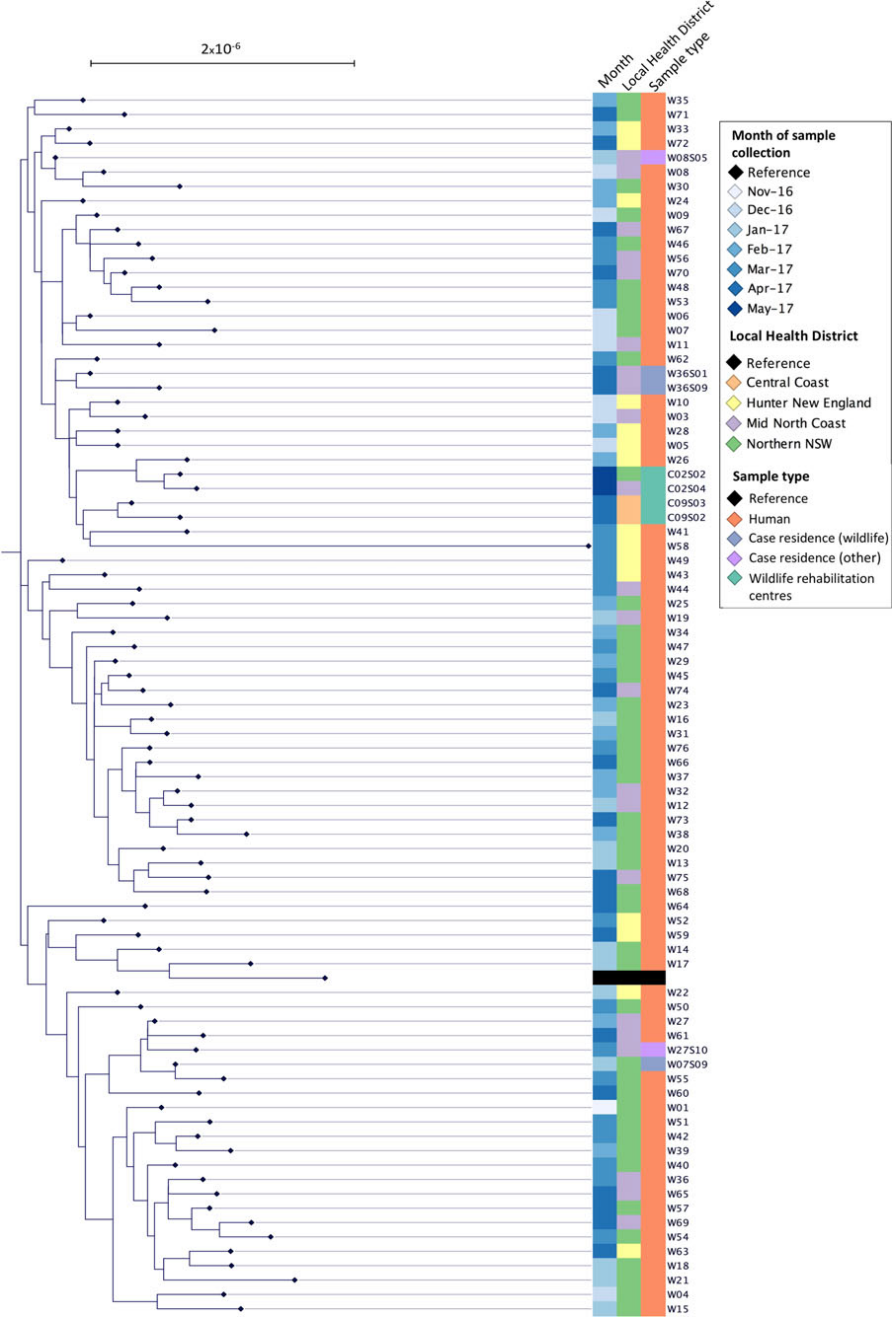
Unrooted maximum-likelihood phylogeny of *S.* Wangata isolates from human cases (*n* = 75) and environmental and animal samples (*n* = 9) collected in Hunter New England, Mid North Coast and Northern NSW Local Health Districts, November 2016 to April 2017. Note: Case residence (other) includes isolates recovered from the environment and animal faeces (exclusive of wildlife) in outdoor areas of cases’ residences.

There were 84 *S.* Wangata isolates sequenced, including isolates obtained from: humans (*n* = 75), case residences (wildlife) (*n* =  3), case residences (other) (*n* = 2) and wildlife rehabilitation centres (*n* = 4). Within the genomes of *S*. Wangata isolates, there were 541 variable sites and 395 core SNPs. Figure 1 shows the phylogenetic SNP tree for all the *S*. Wangata isolates using the assembled *S*. Wangata reference. The average pairwise SNP distance was 5.531 × 1^6^. One isolate (W58) showed greater SNP variation than all others with an average SNP difference of 83. When this sample was excluded, the median SNP difference between all remaining isolates was 24 (range 6–48 SNPs), indicating the remaining isolates were relatively clonal.

There was no distinct clustering by time of isolation or geospatial location (Fig. 1). Of the four cases where *S*. Wangata was recovered from the outdoor environment of cases’ residences, two isolates had a low number of SNP differences in relation to the associated human isolates (seven SNPs (W08 and W08S05 – compost) and eight SNPs (W27 and W27S10 – dog)). By comparison, isolates recovered from wildlife scats in the outdoor environment of cases’ residences showed a greater number of SNP differences (31 SNPs (W07 and W07S09), 22 SNPs (W36 and W36S01) and 32 SNPs (W36 and W36S09)). Wildlife isolates were clustered by rehabilitation centre (Fig. 1).

## Discussion

This is one of the first outbreak investigations of an environmental *Salmonella* serovar in Australia to use both epidemiological and high-resolution genomic data. *S*. Wangata was isolated from the outdoor environments of cases’ residences, as well as from wildlife in rehabilitation centres, providing further evidence that this serovar likely has an environmental source. Indirect exposure to bats/flying foxes, wild frogs and wild birds were identified as risk factors for *S*. Wangata infection in the case–control study; however, samples were not collected from bats/flying foxes or frogs during the investigation and therefore these associations could not be explored through microbiological testing. Further investigation is required to confirm environmental and animal reservoirs.

Whilst bats/flying foxes are known vectors for viral pathogens in Australia, their carriage of bacterial pathogens is less well understood. In a recent study in Melbourne, Australia, *Salmonella* was isolated from two flying fox colonies and a daily load of 4 × 10^6^ organisms was estimated [22]. However, this study was only able to identify *S. enterica* species and was unable to calculate the risk to human health. Further studies examining the bacterial microflora of flying fox faeces to determine the prevalence of *S. enterica* serovars would be valuable.

Wild frogs have been previously associated with indirect transmission of *Salmonella* to humans via contaminated environments and water, particularly in northern tropical areas of Australia [8, 23]. In addition, wild birds can often be found in human-dominated landscapes and may act as an important vector for bidirectional transmission of *Salmonella* in Australia [24]. Our investigation detected *S.* Wangata in waterfowl (pelican and black swan) and a recent study detected *S.* Wangata in a shorebird (silver gull) [25], suggesting that water may play a role in the transmission of this serovar.

Contact with household pets was not identified as a significant risk factor for *S*. Wangata infection in our investigation. However, *Salmonella* was isolated from two pet dog faecal samples collected in the investigation, one of which was serotyped as *S*. Wangata (W27S10) and shared a close phylogenetic relationship with the human case from the same residence (W27). Dogs and other household pets may act as bidirectional vectors for environmental *Salmonella* serovars due to their interactions with both the environment and humans [24]. In our investigation, both dogs from which *Salmonella* was isolated showed no signs of illness; this is consistent with a study in the UK that found healthy dogs can shed *Salmonella* subclinically [26]. Whilst we cannot rule out that the owner and pet dog were both exposed to the same environmental source, transmission of *Salmonella* from household pets to owners has been identified in other studies [27–29] and the potential risk of this transmission pathway should be included in public health messaging.

We did not find an epidemiological association between *S*. Wangata infection and contact with livestock, property size or use of a private water supply. In addition, *Salmonella* was not detected in any of the water samples collected from cases’ residences. These findings are in contrast to previous studies that have implicated drinking water as a vehicle of infection for other *Salmonella* serovars [6, 23]. Outdoor activities and contact with soil or grass were also not identified as significant risk factors in our investigation. However, *Salmonella* serovars Wangata, Birkenhead and Kiambu were identified in soil/compost samples collected from cases’ residences, indicating that contaminated soil may be a plausible transmission pathway in some instances.

We found that the odds of direct exposure to pet chickens, as well as growing nut trees and eating home-grown foods, were significantly lower among *S*. Wangata cases compared with the respective control groups in the multivariable analysis. However, these behaviours are not biologically plausible protective factors for environmental *Salmonella* transmission.

We found *S*. Wangata isolates were relatively clonal overall, with fewer SNP differences between isolates when compared with other environmental *Salmonella* investigations that included isolates from both humans and animals [30, 31]. The average number of SNPs (24) was also lower than the average observed in a recent investigation of sporadic *S*. Enteriditis [32], however the *S*. Enteriditis isolates were obtained over a longer time period. Indeed, the short time period during which samples were collected could have inflated the perceived clonality of these isolates. One theory explaining the clonal nature of isolates could be that the cases were linked via a common wildlife reservoir as recorded in past outbreaks [7, 30]. Wildlife scats collected from the environment of cases’ residences did not appear to have a close phylogenetic relationship with the associated human cases. This may be attributed, in part, to the delay between case illness onset and environmental sampling, during which time the wildlife species in the cases’ environments may have changed. The fact that *S.* Wangata was isolated from wildlife scats in the cases’ environment suggests that wildlife may be a vehicle for transmission in some instances.

There are a number of strengths and limitations in our investigation. Prior to this investigation, there was limited genomic information available on the *S.* Wangata serovar. Through the assembly of an *S*. Wangata isolate, we were able to compare across the whole genome and include regions that may be unique to this serovar. This assembly is an important contribution to future epidemiological and genomic studies on *S.* Wangata.

A further highlight was the strength of multidisciplinary collaboration between partner organisations. By adopting a One Health approach, we were able to better investigate the interactions at the human–animal–environmental interface.

The inclusion of two control groups provided benefits in relation to recall bias (*S*. Typhimurium controls) and representativeness (neighbourhood controls). Having both control groups enabled us to more accurately interpret risk factors for *S*. Wangata infections. Due to low numbers of *S.* Typhimurium notifications during the investigation period, we were only able to obtain small numbers of controls in some age groups. Age was adjusted for in the multivariable model, however statistical precision may have been lost in relation to age due to the small sample. For neighbourhood controls, we applied a self-randomisation method within households (completing the questionnaire for the person with the next birthday). However, we did not collect information on household size or apply weighting in this regard. Our data may have been influenced by single-person households, which reduced the proportion of responses for younger persons (<15 years). Sex was not collected for neighbourhood controls in our investigation. Historically, *S*. Wangata cases have been equally distributed by sex and we found that it was not a significant predictor in the *S.* Typhimurium model.

We encountered some issues in the collection of environmental samples in our investigation. The delay between cases’ onset of illness and the date of environmental sample collection may have reduced our ability to detect *Salmonella*. Major flooding in the north-eastern NSW region during our investigation period reduced access to some residences for sampling and prevented participation by some wildlife rehabilitation centres, thereby reducing the number of environmental samples collected. It is also possible that flooding may have introduced or removed *S*. Wangata contaminates from cases’ residences. In addition, the lack of samples from bats/flying foxes and wild frogs meant we were unable to compare the epidemiological findings with samples from these animals.

In summary, our results indicate that *S*. Wangata may have a reservoir in wildlife populations in north-eastern NSW with important implications for animal and human health in Australia. However, transmission pathways from wildlife to humans remain unclear from epidemiological and genomic analyses. Further research into the occurrence of *Salmonella* among wildlife groups − particularly bats/flying foxes, wild frogs and wild birds − would be useful to enhance our understanding of the sources of *S*. Wangata.

## Appendix 1

Wildlife species sampled during the investigation and Salmonella serovars isolated.

**Table:**

Species	Common name	No. samples	No. positive	Serovar (No. isolated)
Mammal				
*Trichosurus vulpecula*	Brushtail possum	1	0	
*Macropus giganteus*	Eastern Grey kangaroo	5	0	
*Nyctophilus gouldi*	Gould's long-eared microbat	1	1	*S*. Bovismorbificans (1)
*Phascolarctos cinereus*	Koala	9	0	
*Pseudocheirus peregrinus*	Ringtail possum	1	0	
*Petaurus norfolcensis*	Squirrel glider	1	0	
*Wallabia bicolor*	Swamp Wallaby	4	0	
*Vombatus ursinus*	Common Wombat	1	1	*S*. Kottbus (1)
Birds				
*Cygnus atratus*	Black swan	1	1	*S.* Wangata (1)
*Ocyphaps lophotes*	Crested pigeon	2	0	
*Platycercus eximius*	Eastern rosella	2	0	
*Eolophus roseicapilla*	Galah	1	0	
*Dacelo novaeguineae*	Kookaburra	1	0	
*Gymnorhina tibicen*	Magpie	3	1	*S*. Bovismorbificans (1)
*Pelecanus conspicillatus*	Pelican	1	1	*S*. Wangata (1)
*Strepera graculina*	Pied currawong	1	0	
*Ptilonorhynchus violaceus*	Satin bower bird	1	0	
*Cacatua galerita*	Sulphur crested cockatoo	1	0	
*Podargus strigoides*	Tawny frogmouth	3	1	*S*. Chailey (1)
Reptiles				
*Tiliqua scincoides*	Eastern blue tongue lizard	1	0	
*Cyclodomorphus gerrardii*	Pink tongued lizard	1	0	
*Chelodina longicollis*	Eastern long necked turtle	1	0	
*Chelonia mydas*	Green sea turtle	4	2	*S*. Wangata (2)
*Eretmochelys imbricata*	Hawksbill turtle	1	0	
	TOTAL	48	8	
